# Tianeptine modulates synaptic vesicle dynamics and favors synaptic mitochondria processes in socially isolated rats

**DOI:** 10.1038/s41598-021-97186-7

**Published:** 2021-09-07

**Authors:** Ivana Perić, Victor Costina, Snežana Djordjević, Peter Gass, Peter Findeisen, Dragoš Inta, Stefan Borgwardt, Dragana Filipović

**Affiliations:** 1 Department of Molecular Biology and Endocrinology, “VINČA”, Institute of Nuclear Sciences - National Institute of the Republic of Serbia, University of Belgrade, Belgrade, Serbia; 2 Institute for Clinical Chemistry, Medical Faculty Mannheim of the University of Heidelberg, University Hospital Mannheim, 68159 Mannheim, Germany; 3 Poisoning Control Centre, Military Medical Academy, Belgrade, Serbia; 4 Department of Psychiatry and Psychotherapy, Central Institute of Mental Health, Medical Faculty Mannheim, Heidelberg University, 68159 Mannheim, Germany; 5 Department of Psychiatry (UPK), University of Basel, Basel, Switzerland; 6 Department of Psychiatry and Psychotherapy, University of Lübeck, Lübeck, Germany

**Keywords:** Biochemistry, Neuroscience

## Abstract

Deregulation of synaptic function and neurotransmission has been linked with the development of major depression disorder (MDD). Tianeptine (Tian) has been used as antidepressant with anxiolytic properties and recently as a nootropic to improve cognitive performance, but its mechanism of action is unknown. We conducted a proteomic study on the hippocampal synaptosomal fractions of adult male Wistar rats exposed to chronic social isolation (CSIS, 6 weeks), an animal model of depression and after chronic Tian treatment in controls (nootropic effect) and CSIS-exposed rats (lasting 3 weeks of 6-week CSIS) (therapeutic effect). Increased expression of Syn1 and Camk2-related neurotransmission, vesicle transport and energy processes in Tian-treated controls were found. CSIS led to upregulation of proteins associated with actin cytoskeleton, signaling transduction and glucose metabolism. In CSIS rats, Tian up-regulated proteins involved in mitochondrial energy production, mitochondrial transport and dynamics, antioxidative defense and glutamate clearance, while attenuating the CSIS-increased glycolytic pathway and cytoskeleton organization proteins expression and decreased the expression of proteins involved in V-ATPase and vesicle endocytosis. Our overall findings revealed that synaptic vesicle dynamics, specifically exocytosis, and mitochondria-related energy processes might be key biological pathways modulated by the effective nootropic and antidepressant treatment with Tian and be a potential target for therapeutic efficacy of the stress-related mood disorders.

## Introduction

The onset of major depression disorder (MDD) in humans is related with chronic social stress; hence, the related paradigms have been developed for modeling psychiatric disorders in animals. Chronic social isolation (CSIS) is a mild psychosocial stress which in rats causes behavioral abnormalities and structural and functional alterations in brain regions^1–6^, including hippocampus^7–14^. Recent studies relate MDD with altered energy metabolism and expression of synaptic proteins as well as mitochondrial dysfunction^15,16^. The alterations in neurotransmission processes mediated via serotonin, dopamine and gamma-aminobutyric acid (GABA), as well as the reduction in synapse number and their functionality have also been associated with MDD. Biochemical changes in the synapses, which arise in response to electrical activity, modify the brain in response to behavior and could represent a significant target for therapeutics dealing with cognitive illnesses^17^. Therefore, in order to understand the organization of the synaptic machinery in health and disease^18^ intensive research in the field of MDD is increasingly focused on the characterization of synaptosomal proteins, which is an artificial structure constructed from neural terminals, including mitochondria, synaptic vesicles and postsynaptic density^17,19^. In spite of the efforts, the involvement of hippocampal synapses with the underlying pathophysiology of MDD, as well as the treatment remain elusive.

Despite wide number of available medications, treating MDD is still challenging. In contrast to the mechanism of most commonly prescribed antidepressants from the class of selective serotonin reuptake inhibitors (SSRIs)^20^, the atypical antidepressant tianeptine (Tian) acts by increasing the reuptake of serotonin from the synapse^21,22^. More lately, Tian’s antidepressant and anxiolytic activities^23,24^ have been related with the modulation of glutamatergic neurotransmission^25,26^. Its efficacy against depressive episodes is comparable with SSRIs, such as fluoxetine, even in patients resistant to SSRI antidepressants therapy^24^. Tian is generally well tolerated by patients and causes less side effects compared to SSRI’s^26^. It promotes neuroplasticity by increasing the expression of neuroplasticity factor genes^27^, reverses stress-induced dendritic atrophy in the hippocampus^23^ and prevents impairments induced by chronic stress on cognitive process such as learning and memory^28,29^. We recently demonstrated that chronic Tian treatment enhances GABA-mediated neurotransmission in the hippocampus of rats exposed to CSIS^12^. Its mechanism of antidepressant and anxiolytic action was also related with its μ-opioid receptor agonist activity^30^. Acting as a positive allosteric modulator of the α-amino-3-hydroxy-5-methy-l-4-isoxazole propionate (AMPA)-type glutamate receptor (AMPAkine), Tian prolongs and strengthens synaptic transmission^31^. Hence, it is considered as one of the nootropics for improving cognitive performances in healthy individuals within hours^32^. Unlike this immediate effect, Tian exerts antidepressant and anxiolytic properties only after chronic treatment. Finding the pathways activated upon chronic Tian administration will contribute to better understanding of its antidepressant action as well as the effects of its prolonged use as a nootropic.

In this study, we performed a comparative proteomics with synapse membrane-enriched (synaptosome) hippocampal fractions of adult male Wistar rats exposed to CSIS (6 weeks) and after chronic Tian treatment of controls (3 weeks) and CSIS-exposed rats (lasting 3 weeks of 6-week-CSIS). Our aim was to profile synaptosomal sub-proteome changes representative of possible time-consuming events underlying the CSIS-induced depressive- like behavior and antidepressant property of Tian and to identify the activated signaling pathways affected by CSIS or Tian treatment. The proteins representative of potentially most altered signal pathways were further validated with Western blot or immunohistochemical analysis. The results of this study may be helpful in identification of deregulated pathways involved in MD^33^ and highlight possible routes of Tian’s nootropic and antidepressant activity.

## Materials and methods

### Animals

Adult male Wistar rats (2.5 months old, weighting 300–400 g) were provided by Animal Facility of “VINČA” Institute of Nuclear Sciences -National Institute of the Republic of Serbia, University of Belgrade. They were housed under standard conditions in groups up to four per cage at a temperature 20 ± 2 °C, humidity 55 ± 10% with access to water and food (commercial rat pellets) ad libitum. All animals were maintained under a 12 h light/dark cycle (lights on 07.00 to 19.00 h). All experimental procedures were approved by the Ethical Committee for the Use of Laboratory Animals of „VINČA" Institute of Nuclear Sciences—National Institute of the Republic of Serbia, University of Belgrade, which follows the guidelines of the EU registered Serbian Laboratory Animal Science Association (SLASA). The study protocol was approved by the Ministry of Agriculture, Forestry and Water Management—Veterinary Directorate, ethics committee, license 323–07-01,893/2015–05. Moreover, rats were monitored on a daily basis. All methods are reported following the recommendations of ARRIVE guidelines.

### Study design

**Figure 1:**
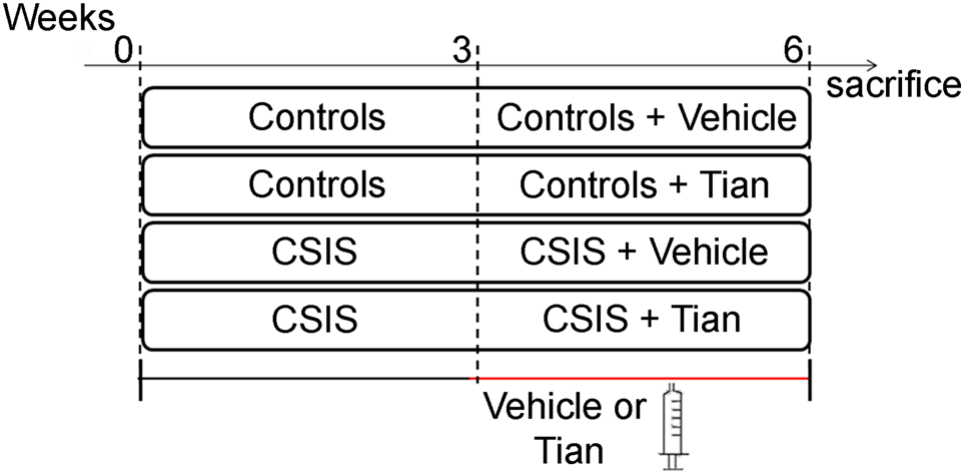
Schematic representation of the study design. CSIS-chronic social isolation. Tian-tianeptine.

For proteomics experiments, rats (n = 40) were divided in two separate batches, with half of the animals being control rats (housed in groups up to four) while the rest were exposed to CSIS (rats housed individually and deprived of any visual or tactile contacts with other animals, but with normal auditory and olfactory experiences^34^. The 6-week experiment was divided into two parts (Fig. 1). During the first 3 weeks, rats were housed under mentioned conditions with no additional experimental procedures. During the second 3 weeks of experiment, half of each batch of animals (Controls and CSIS) were treated with intraperitoneal (ip) injections of Tian solution (10 mg/kg/day) (Control + Tian and CSIS + Tian), while the rest were treated with vehicle (Veh, physiological saline) (Control + Veh and CSIS + Veh). The assessment of depressive- and anxiety-like behaviors in rats, and the exclusion of rats resilient to CSIS or Tian treatment, was performed according to the results of behavior parameters in sucrose preference^35^, marble burying^36^ and forced swim test^37^, which was previously published^14^. Briefly, in sucrose preference (SP) test we measured the percentage preference of the rats for 2% sucrose solution compared to tap pure tap water after 1 h, whereby a decline in SP was indicative of anhedonia, as a core symptom of MDD. In marble burying (MB) test, we recorded the number of buried marbles after 30 min, whereby an increase in the number of buried marbles was indicative of anxiety-like behavior. In the forced swim test (FST), the time of immobility behavior of the rats measured during 5 min period of test duration, was indicative of behavioral despair in rats. The reversal effects of Tian in all three performed tests were considered as the antidepressant- and anxiolytic-like effects of the drug. The last behavioral testing was carried out 24 h before sacrification procedure.

The final number of animals per group was n = 6.

### Preparation of Tianeptine solution

To prepare Tianeptine sodium solution (Tian) Coaxil tablets (Les Laboratoires Servier Industrie, France) were crushed and dissolved in physiological saline with the aid of ultrasound and subsequently filtered through Whatman No. 42 filter paper and Millipore Express PES membrane filter (0.22 µm). The actual concentration of Tian in solution (3.94 mg/ml) was determined with the use of high-performance liquid chromatography with photodiode array detector. Tian was administered (10 mg/kg/day)^27,38^ according to rats’ weights that were measured once a week. This dosage was based on indications of literature reviews documenting its efficacy in attenuating stress-induced behavioral and neurochemical abnormalities in a chronic setting^39,40^. Further, Tian administration for 3 weeks resulted in serum concentrations of 12 ± 1.15 ng/ml in Tian-treated controls and 13.40 ± 0.81 ng/ml in Tian-treated CSIS rats. The analysis was performed with the use of LC/MS in serum samples obtained 24 h after the last Tian administration.

### Preparation of synaptosomal fractions

The animals were deeply anesthetized with ip injections of ketamine/xylazine (100/5 mg/kg), perfused with physiological saline and sacrificed by guillotine (Harvard Apparatus, South Natick, MA, USA) decapitation. Brain was removed, hippocampus was dissected on ice, shock-frozen in liquid nitrogen, and stored at—80 °C until further analyses. Within each group, both rat hippocampal hemispheres of two hippocampi were pooled and homogenized in cold homogenization buffer (10 mM Tris/HCl (SERVA) pH 7.4, 0.25 M sucrose (Fisher Scientific)) containing protease inhibitor cocktail tablet (complete tablets, Mini, EASY pack, Roche) by 10 strokes at 800 rpm, in the Potter–Elvehjem homogenizer with Teflon pestle. The removal of nucleus fraction was performed by centrifugation at 1300 × g for 10 min. Supernatant was further re-centrifuged under the same conditions for removal of the remained nuclei. From nuclei-free supernatant, total mitochondria and synaptosome fractions were separated in the form of pellet by centrifugation on 19,200 × g at 4 °C for 15 min (Sorvell Beckman, JA-20). The pellet containing mitochondria and synaptosomes was processed based on Percoll (GE Healthcare) gradient procedure (15%, 24%, and 40%) in sucrose buffer^41^, for separation of synaptosomal fraction. After centrifugation (37,000 × g, 15 min in a Beckman L8-M Ultracentrifuge Ti50), the separated fractions of synaptosomes (the contact surface between 15 and 24% Percoll layers) and nonsynaptic mitochondria (NSM) (the contact surface between 24 and 40% Percoll layers) were carefully collected, washed twice in ten volumes of homogenization buffer and further centrifuged at 14,000 × g for 30 min at 4 °C. Both, synaptosomes and NSM were lysed in buffer (5 mM Tris–HCl pH 8.1, 0.5 mM EDTA) and homogenized using glass pestle tissue homogenizer for one minute. All collected fractions were stored at—80 °C until the use. The relative purity of isolated subcellular fractions was confirmed by the absence of nuclear/cytosolic contaminations of the synaptosomal fractions after incubation of control samples with antibody against nuclear (anti-TATA binding protein), cytosolic (anti-α tubulin) and synaptosol (anti-synaptophysin) proteins, demonstrated in our previous study^14^. The protein concentrations were measured by the method of Lowry^42^ using purified bovine serum albumin as a standard.

### Electrophoresis and mass spectrometry

Electrophoretic separation of the synaptosomal fractions as well as mass spectrometry analysis were performed as previously described^43^. Extracted MS/MS spectra were searched against the Uniprot/Swissprot database using the Proteome Discoverer (version 1.3) search engine (Thermo Fisher Scientific) accepting common variable modifications and one missed tryptic cleavage. Peptide tolerance was ± 10 ppm and MS/MS tolerance was ± 0.5 Da. All protein identification experiments were carried out using the corresponding decoy database and a false discovery rate (FDR) of 1%. The relative quantification of the proteins in the two subgroups were performed with the label free quantification (LFQ) tool of the Sieve 2.0 software (Thermo Fisher Scientific) using a mass error tolerance of ± 10 ppm and a retention time shift of ± 1 min. The mass spectrometry proteomics data have been deposited to the ProteomeXchangeConsortium via the PRIDE partner repository^44^with the dataset identifier PXD021920.

### Immunohistochemistry

We performed immunohistochemical (IHC) procedure for validation of observed Synapsin-1 (Syn1) expression changes upon CSIS and/or Tian treatment. Separate batch of animals (n = 4–6 per group) was used for IHC validation purpose. Animals were prepared as described in the “Study design” section. Upon sacrification, whole brains were immediately isolated and kept in 4% paraformaldehyde (PFA) in phosphate-buffered saline (PBS) at 4 °C during the night. Subsequently, brains were transferred into 0.4% PFA solution. Brains were cut into 40 µm-thick coronal sections with a vibratome (VT 100 S; Leica Bensheim, Germany). Prepared sections were kept in cryoprotectant solution (15% glucose, 30% ethylene glycol in 0.05 M PBS, pH 7.4) at -20 °C until the staining procedure. Briefly, sections (Bregma -3.12 to -3.60 mm, according to Paxinos and Watson^45^) were rinsed three times in PBS containing 0.05% Triton and subsequently incubated with 0.6% hydrogen-peroxide in PBS with Triton for 30 min. Afterwards, sections were blocked in 2% normal goat serum for 1 h and incubated during the night at 4 °C with primary antibody against Syn1 (Cell Signaling, D12G5) diluted (1:500) in blocking solution. The next day, after being washed in PBS, sections were incubated with biotinylated anti-rabbit secondary IgG antibody (Vector laboratories, 1:500 in PBS) for 1 h. We incubated the sections in ABC kit (VECTASTAIN, Vector laboratories) for 20 min. The staining of the immunohistochemical complexes was performed with 3,3'-diaminobenzidine (D5637 Sigma-Aldrich). Sections were mounted on a gelatin-coated glass slides and cover-slipped with Eukitt medium. The pictures were taken on a Zeiss microscope equipped with camera AxioCAMERc 5S. We analyzed the Syn1 expression change by densitometric analysis with the use of Image J software (version 1.52i). The average value was conducted of ten independent densitometric measurements per section (n = 2–3) for each biological replicate.

### Western blotting

The validation of the synaptoproteomic data for selected proteins was performed by Western blot on a separate batch of animals (n = 4–6 per group) that underwent the same procedure as explained in the “Study design” section. Prior the hybridization with primary antibodies, membranes were cut at desirable range of protein masses (kDa) based on the Thermo Scientific PageRuler Plus Prestained Protein Ladder (#26,619). We used anti-α-tubulin (sc-5286, Santa Cruz, 1:1000) (detected in the part of membrane between 50–72 kDa), anti-cytochrome c (Cyt c) (Santa Cruz, sc-13156, 1:1000) (detected in the part of membrane between 0–35 kDa), anti-HSP90 (sc-13119, Santa Cruz, 1:200) (detected in the part of membrane between 72–250 kDa), anti-glutamate dehydrogenase 1 (Glud1) (ARP45709, Aviva Systems Biology, 1:1000) (detected in the part of membrane between 50–72 kDa) and anti-glutamine synthetase (Glul) (376,767, Santa Cruz, 1:100) (detected in the part of membrane between 35–50 kDa), followed by incubation with HRP-conjugated IgG antibodies (anti-mouse (Sigma Aldrich, A9917, 1:10,000) or anti-rabbit (Sigma Aldrich, A9169, 1:30,000)) diluted in TBST (Tris Buffered Saline with Tween 20). Band intensities were normalized against Ponceau S staining^46^. The chemiluminescent signal was induced with Immobilion Western, Chemiluminescent HRP Substrate (Millipore, USA), detected with Chemidoc-MP System (Bio-Rad) and analyzed with Image Lab 5.0 software (Bio-Rad). Ponceau S staining intensity was analyzed with the use of ImageJ software (Version 1.52i).

### Bioinformatics and statistical analysis

Interactome study and assignation of the biological processes and molecular functions of the identified proteins were performed with the use of STRING (version 11.0). Proteomic data are presented according to software pre-set p < 0.01 for peptides and p < 0.05 for proteins. Only proteins showing protein fold changes higher than 1.2 and lower than 0.8 have been considered for further analysis. Proteins and peptides identified according to only one peptide match and/or one unique peptide were excluded from bioinformatics analysis.

Western blot and immunohistochemical analysis data have shown normal distribution according to Shapiro–Wilk test and hence were analyzed with the use of parametric test, a two-way ANOVA. The analyses were performed with Statistica 12 software. The significance level was set at p < 0.05. The results were presented as average ± SEM.

## Results

### Differential protein analysis of synaptosomal fractions

The entire lists of common identified proteins in the hippocampal synaptosomal fractions with fold changes are provided in the Supplementary Tables S1–S3. Differential proteomic results in Controls treated with Tian resulted with 73 proteins up-regulated (Supplementary Table S1). Tian increased the expression of several proteins associated with vesicle-mediated transport such as Dnm1, Dpysl2, Hspa8, Pacsin1, Rab3a, Stx1b, Stxbp1, Syt1. Also, Syn1 and Rab proteins, related with the synaptic processes, together with proteins involved in cytoskeleton organization (Crmp1, Dpysl2, Pacsin1, Sptan1, Tuba1c, Tuba4a, Tubb3) were found up-regulated. Beside vesicle-mediated proteins, Tian increased the expression of a considerable number of enzymes involved in glycolysis, such as Aldoa, Aldoc, Eno3, Hk1, Pgam1, Pkm, RGD1560402 and glyceraldehyde-3-phosphate dehydrogenase (Gapdh). Mitochondrial energy metabolism-related proteins Atp5b, Atp5o, Atp1a1, Atp1a2, Atp6v0a1, Atp6v1b2, Atp6v1e1 were also found up-regulated. CSIS vs. Controls showed 118 up-regulated proteins (Supplementary Table S2). The majority of up-regulated proteins were enzymes involved in glucose metabolism (Aldoa, Aldoart2, Aldoc, Eno3, Gpi, Pdhb, Pgam1, Pgk1, Pkm) and tricarboxylic acid (TCA) (MDH2). The expression of proteins involved in regulation of signal transduction (Camk2d, Gdi1, Gnai2, Gnai2, Mapk1, Pebp1, Ppp3ca, Prkcg, Rab2a, Rab3a, Rhob), synaptic transmission (Snap25, Stx1b, Stxbp1, Syn1, Syn2, Syt1, Syt2) and vesicle-mediated transport in synapse (Dnm1, Dpysl2, Rab3a) was up-regulated. V-ATPase proteins (Nsf, Atp4a, Atp6v0d1, Atp6v1a, Atp6v1b2, Atp6v1e1, Atp6v1h) were also up-regulated. CSIS induced an increase in the Ap2a2, which is a part of the AP-2 complex. A total of 78 proteins were differentially expressed, with 30 up- and 48 down- regulated, respectively, from the matches between CSIS + Tian vs. CSIS (Supplementary Table S3. TCA cycle proteins (Dlat, Pdhb, Pdha1/1) and subunits of proteins involved in oxidative phosphorylation (Ndufs3, Uqcrc2, Cyc1, Atp5a1, Atp5c1, Taf3) were found up-regulated. Like in Controls, Tian up-regulated the proteins involved in vesicle-mediated transport (Rab2a, Rab3a, Rab33b, Rab7a, Rala, Ywhaz, Vesicle-associated membrane protein 2). Regulatory proteins, such as chaperone proteins Hspe1 and Hspa9, as well as Prdx5, Sod_Cu domain-containing protein and Gsto1, were up-regulated. Particularly interesting is an increase in myo-inositol phosphatase (Impa1) level in CSIS and additionally boosted in the Tian-treated CSIS rats.

Overlapping proteins between CSIS vs. Controls and CSIS + Tian vs. CSIS as well as Tian vs. Controls and CSIS + Tian vs. CSIS are presented in Tables 1 and 2, respectively. These data show an insight into overlapping proteins being deregulated by CSIS while also being de- or re-regulated by Tian treatment. In Table 1, 43 proteins were up-regulated in CSIS vs Controls, while 10 proteins were up- and 33 proteins were down-regulated in CSIS + Tian vs. CSIS. Regarding Tian treatment of Controls and CSIS rats, 31 proteins were up-regulated, whereby 5 proteins were up- and 26 proteins were down-regulated by Tian in CSIS (Table 2).

**Table 1:** The list of differently expressed synaptosomal proteins between CSIS versus Control and CSIS + Tian versus CSIS. CSIS-chronic social isolation, Tian-tianeptine.

Protein name	UniProtKB accession no	Gene	Ratio	Ratio
			CSIS versus Control	CSIS + Tian versus CSIS
Inositol-1-monophosphatase	F1M978	Impa1	3.86	1.35
Phosphoglycerate mutase 1	P25113	Pgam1	3.77	2.14
RAB33B, member RAS oncogene family	F1LW77	Rab33b	2.54	1.76
Vesicle-associated membrane protein 2	Q19LA7	N/A	2.34	1.85
Ras-related protein Rab-2A	F1LP82	Rab2a	2.23	1.96
Glutamate dehydrogenase 1, mitochondrial	P10860	Glud1	2.07	1.32
Uncharacterized protein	A0A0G2K099	N/A	2.00	1.34
Pyruvate dehydrogenase E1 component subunit beta	A0A0G2KAM3	Pdhb	1.93	1.50
Ras-related protein Rab-3A	P63012	Rab3a	1.89	1.41
Pyruvate dehydrogenase E1 component subunit alpha	D4A5G8	Pdha1l1	1.60	1.45
Heat shock protein 86	Q6B437	N/A	5.21	0.72
Glucose-6-phosphate isomerase	Q6P6V0	Gpi	4.90	0.66
Clathrin coat assembly protein AP180	F1LRK0	Snap91	4.22	0.39
ATPase H + -transporting V1 subunit A	A0A1W2Q6N0	Atp6v1a	4.00	0.65
Endophilin-A1	O35179	Sh3gl2	3.99	0.60
Dynamin-1	P21575	Dnm1	3.89	0.63
AP-2 complex subunit alpha	A0A0G2K943	Ap2a2	3.63	0.50
Fructose-bisphosphate aldolase	A0A0G2K3Q6	Aldoc	3.52	0.68
Beta-enolase	P15429	Eno3	3.51	0.71
Phosphoglycerate kinase	M0R6Y8	RGD1560402	3.46	0.71
Alpha-1,4 glucan phosphorylase	G3V6Y6	Pygb	3.43	0.76
Pyruvate kinase	A0A0G2JVG3	Pkm	3.30	0.75
Aspartate aminotransferase, cytoplasmic	P13221	Got1	3.29	0.78
Protein kinase C and casein kinase substrate in neurons protein 1	A0A0G2JWR2	Pacsin1	3.23	0.74
Aminopeptidase	F1M9V7	Npepps	3.20	0.67
Guanine deaminase	Q9JKB7	Gda	3.19	0.75
cAMP-dependent protein kinase type II-beta regulatory subunit	A0A0G2K5G0	Prkar2b	3.12	0.64
Tubulin alpha chain	A0A0H2UHM7	LOC100909441	3.07	0.59
Tubulin beta-2A chain	P85108	Tubb2a	3.06	0.62
Tubulin beta-5 chain	P69897	Tubb5	3.00	0.63
Serine/threonine-protein phosphatase 2B catalytic subunit alpha isoform	P63329	Ppp3ca	2.83	0.69
Septin 7	D4A0F5	Sept7	2.83	0.75
Tubulin beta chain	G3V7C6	Tubb4b	2.74	0.71
Septin 5	D3ZT07	Sep5	2.61	0.79
Mitogen-activated protein kinase 1	P63086	Mapk1	2.53	0.74
Spectrin alpha chain, non-erythrocytic 1	A0A0G2K1Y8	Sptan1	2.44	0.73
ATPase, H + transporting, V1 subunit E isoform 1, isoform CRA_a	G3V7L8	Atp6v1e1	2.39	0.66
Vesicle-fusing ATPase	A0A0G2K6U1	Nsf	2.32	0.64
Contactin-1	Q63198	Cntn1	2.11	0.75
Syntaxin-binding protein 1	P61765	Stxbp1	1.99	0.77
Spectrin beta chain	G3V6S0	Sptbn1	1.96	0.75
Tenascin-R	F1LQ63	Tnr	1.84	0.58
Neurofascin	D3ZW56	Nfasc	1.59	0.71

Proteins were identified according to MS/MS spectra search against the Uniprot/Swissprot database using the Proteome Discoverer (version 1.3) search engine (Thermo Fisher Scientific).

**Table 2:** The list of differently expressed synaptosomal proteins between Control + Tian versus Control and CSIS + Tian versus CSIS.

Protein name	UniProtKB accession no	Gene	Fold change	Fold change
			Control + Tian versus Control	CSIS + Tian versus CSIS
Phosphoglycerate mutase 1	P25113	Pgam1	3.99	2.14
14–3-3 protein zeta/delta	A0A0G2JV65	Ywhaz	3.41	2.06
Ras-related protein Rab-3A	P63012	Rab3a	2.52	1.41
Rat lipophilin	Q63436	N/A	2.04	1.22
RAB33B, member RAS oncogene family	F1LW77	Rab33b	2.01	1.76
Glyceraldehyde-3-phosphate dehydrogenase	D3ZGY4	N/A	4.52	0.76
Alpha-1,4 glucan phosphorylase	G3V6Y6	Pygb	3.91	0.76
Beta-enolase	P15429	Eno3	3.71	0.71
Dynamin-1	P21575	Dnm1	3.18	0.63
Clathrin heavy chain	F1M779	Cltc	3.01	0.51
Spectrin alpha chain, non-erythrocytic 1	A0A0G2K1Y8	Sptan1	2.58	0.73
Tubulin alpha chain	A0A0H2UHM7	LOC100909441	2.52	0.59
Spectrin beta chain	G3V6S0	Sptbn1	2.50	0.75
Contactin-1	Q63198	Cntn1	2.46	0.75
Tenascin-R	F1LQ63	Tnr	2.46	0.58
Vesicle-fusing ATPase	A0A0G2K6U1	Nsf	2.44	0.64
Fructose-bisphosphate aldolase	A0A0G2K3Q6	Aldoc	2.40	0.68
Beta-actin	A0A068F1Y2	Actb	2.33	0.80
ATPase H + -transporting V1 subunit A	A0A1W2Q6N0	Atp6v1a	2.28	0.65
Dephosphin long form	Q7M077	N/A	2.20	0.23
Neurofascin	D3ZW56	Nfasc	2.16	0.71
Aspartate aminotransferase, cytoplasmic	P13221	Got1	2.15	0.78
Syntaxin-binding protein 1	P61765	Stxbp1	2.09	0.77
Septin 5	D3ZT07	Sept5	2.04	0.79
Serine/threonine-protein phosphatase 2B catalytic subunit alpha isoform	P63329	Ppp3ca	2.03	0.69
Tubulin alpha-4A chain	Q5XIF6	Tuba4a	2.00	0.56
Mitogen-activated protein kinase 1	P63086	Mapk1	2.00	0.74
Protein kinase C and casein kinase substrate in neurons protein 1	A0A0G2JWR2	Pacsin1	1.84	0.74
Phosphoglycerate kinase	M0R6Y8	RGD1560402	1.66	0.71
ATPase, H + transporting, V1 subunit E isoform 1, isoform CRA_a	G3V7L8	Atp6v1e1	1.28	0.66
Guanine deaminase	Q9JKB7	Gda	1.24	0.75

CSIS-chronic social isolation, Tian-tianeptine. Proteins were identified according to MS/MS spectra search against the Uniprot/Swissprot database using the Proteome Discoverer (version 1.3) search engine (Thermo Fisher Scientific).

### Protein–protein interaction – STRING

STRING 11.0 software was used for the identification of protein interactions, as well as biological and molecular functions and KEGG pathway assignment regarding all differently expressed proteins.

**Figure 2:**
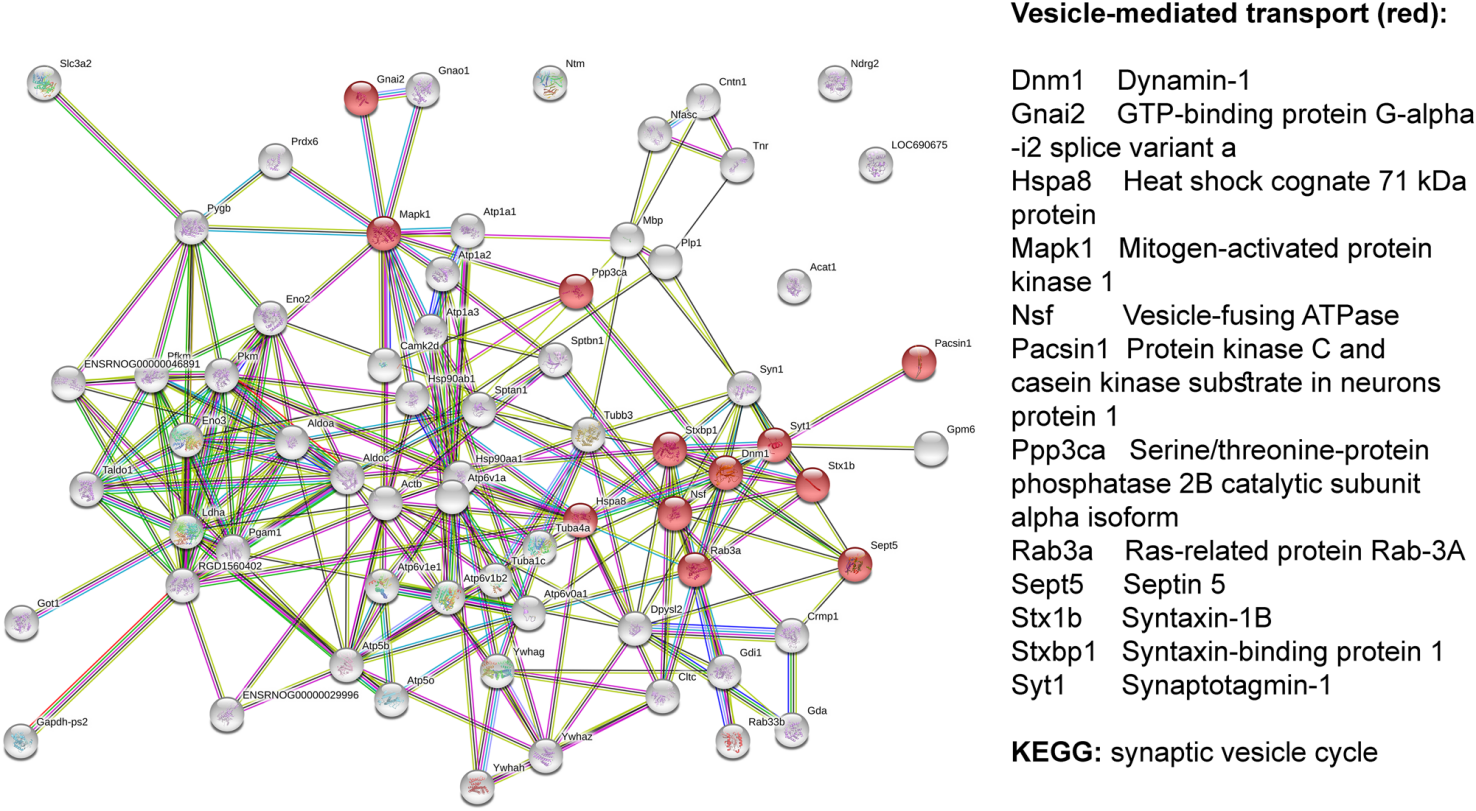
STRING-based interactome map of interactions among differently up-regulated synaptosomal proteins in Tian-treated Controls with, the corresponding GO Biological process terms.

For Tian-treated Controls, significantly more interactions were revealed among up-regulated proteins with enrichment (p < 1 × 10^–16^). Tian over-expressed the proteins related to regulation of transport, specifically vesicle-mediated transport, ATP metabolic process, while synaptic vesicle cycle and glycolysis/gluconeogenesis were recognized as significantly changed pathways by KEGG tool (Fig. 2).

**Figure 3:**
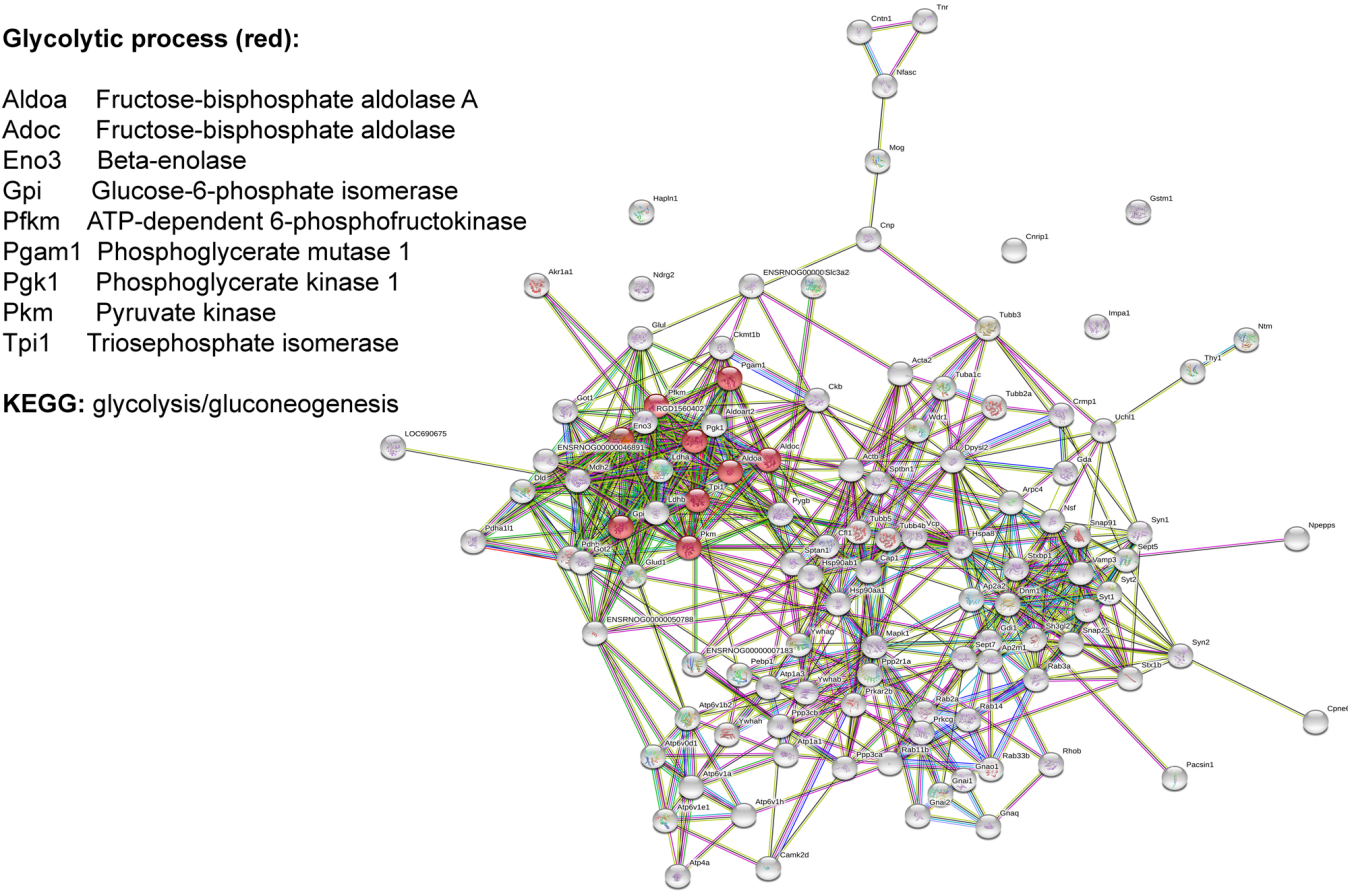
STRING-based interactome map of interactions among differently up-regulated synaptosomal proteins in CSIS vs. Control rats, with the corresponding GO Biological process term.

According to STRING, protein changes in the CSIS rats showed significant interactions among up-regulated proteins, with enrichment p < 1 × 10^–16^. Up-regulated proteins participate mainly in the transport i.e. vesicle-mediated transport, metabolic process, among which the glycolytic process too, and organelle organization (Fig. 3). Protein, ion, and chaperone binding as well as catalytic activity as molecular functions, were found. KEGG pathways found as significant were glycolysis/gluconeogenesis, synaptic vesicle cycle and phagosome.

**Figure 4:**
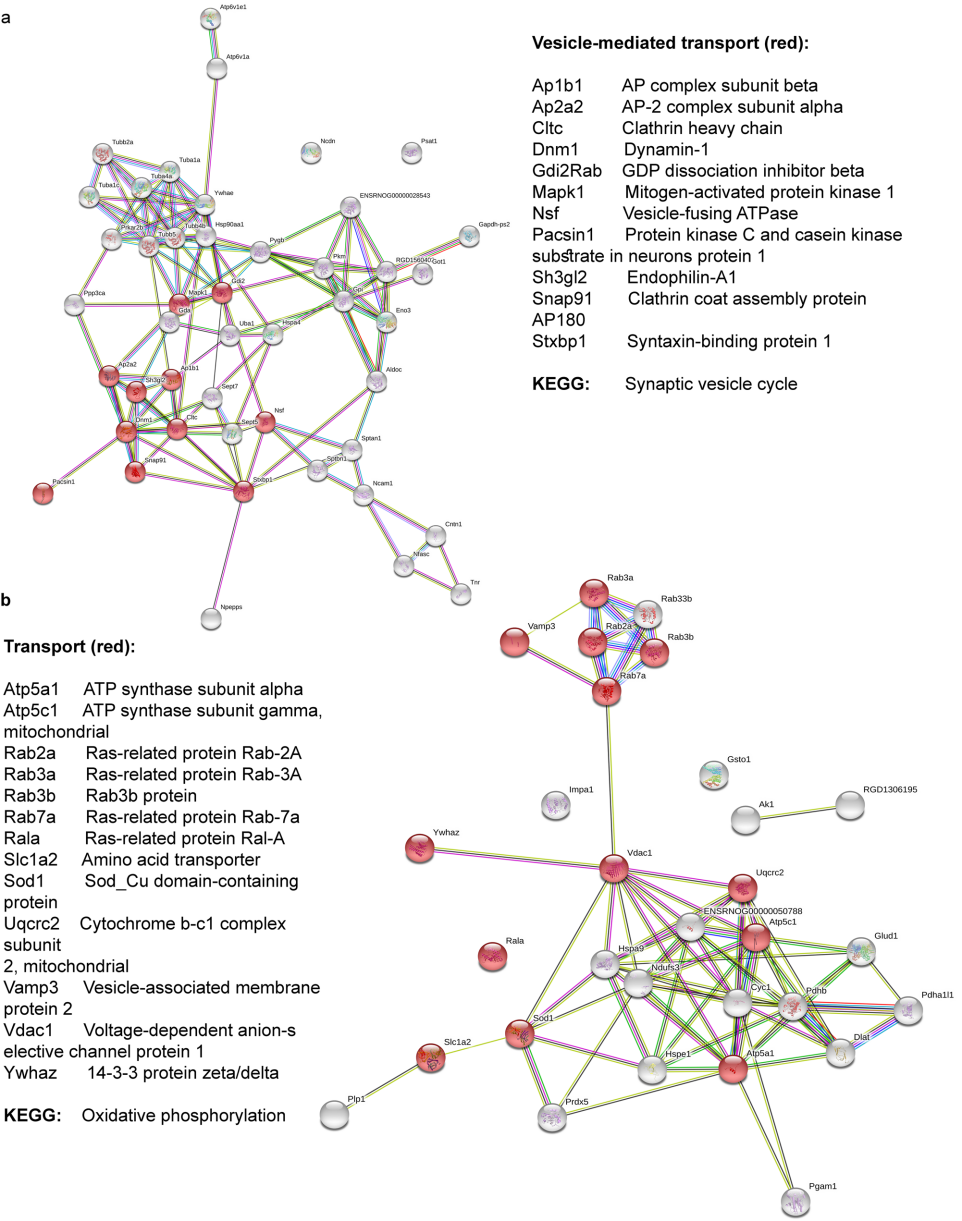
STRING-based interactome map of interactions among differently regulated proteins in Tian-treated CSIS rats, with the corresponding GO Biological process terms: (**a**) up-regulated synaptosomal proteins and (**b**) down-regulated synaptosomal proteins.

For the protein change observed in Tian-treated CSIS compared with CSIS alone, STRING indicated significant interaction among down- (1 × 10^–16^) and up-regulated proteins (p < 1 × 10^–16^). Up-regulated proteins were mainly involved in transport, TCA cycle, oxidation–reduction process and phosphate containing compound. The activated pathway by KEGG was oxidative phosphorylation (Fig. 4a). Among main biological processes affected by down-regulated proteins were transport, mainly vesicle mediated and protein transport, cytoskeleton organization with synaptic vesicle cycle and phagosome activated KEGG pathways (Fig. 4b).

### Immunohistochemical validation of the Syn1 expression changes

**Figure 5:**
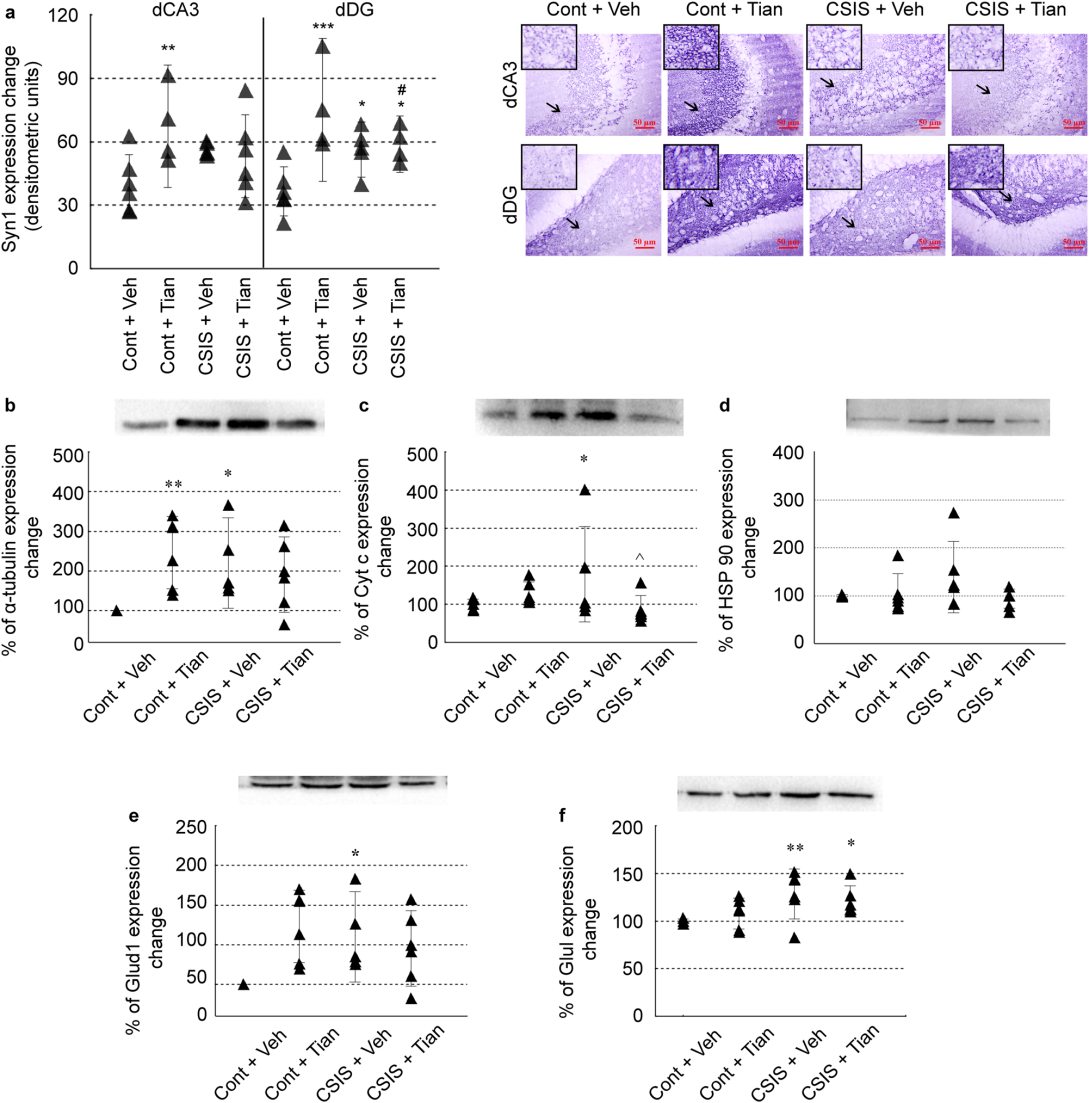
Validation of synapsin-1 (Syn1), α-tubulin, cytochrome c (Cyt c), HSP90, glutamate dehydrogenase 1 (Glud1) and glutamine synthetase (Glul) in synaptosomal fractions of rat hippocampus from Controls, Control + Tian, CSIS and CSIS + Tian groups by Immunohistochemical (**a**) and Western blot analysis (**b**–**f**). Data were expressed as mean ± SEM, n = 4–6 independent measurements in each group. Significant differences between groups obtained from two-way ANOVA analyses followed by Duncan’s post-hoc test are indicated as follows: *p < 0.05, **p < 0.01, comparisons always against Controls; ^p < 0.05, CSIS + Tian vs. CSIS. Displayed blots are cropped images of representative examples of several Western blots performed. Full-length blots are presented in Supplementary Figure S1. The missing parts of the membranes are due to unsatisfactory antibody hybridization, and thus were not represented or used in the final analysis.

Syn1 expression change was validated with the use of immunohistochemical procedure in the coronal sections of the dorsal (d) hippocampus. The significant main effect of combined impact of Tian × CSIS was seen in the dCA3 subregion of the hippocampus (F_1.17_ = 4.55, p < 0.05). Tian significantly increased the Syn1 expression in the dCA3 of Controls (1.69 fc) (^**^p < 0.01). The same trend was seen in the CSIS rats (1.42 fc) and Tian-treated CSIS rats compared to Controls, however no statistical significance was observed. Also, no difference was observed by comparing Tian- and vehicle treated CSIS rats (0.94 fc). Similar pattern of expression changes was revealed in the dDG hippocampal subregion. A significant main effect of Tian (F_1.16_ = 16.30, p < 0.001) and a combined effect of Tian × CSIS (F_1.16_ = 12.57, p < 0.01) in the dDG was revealed by ANOVA. Tian and CSIS caused a significant increase in the Syn1 expression (2.15 fc (^***^p < 0.001) and 1.61 fc (^*^p < 0.05), respectively), while no difference was observed in the expression changes following Tian treatment of the CSIS rats compared to CSIS (1.04 fc). These data are in accordance with the results obtained from the proteomic study. The results of the validation procedure are presented in Fig. 5a.

### Western blotting validation of selected differentially expressed proteins

To validate proteomic results, we selected several candidate proteins, namely, α-tubulin, Cyt c, HSP90, Glud1 and Glul for validation by Western blot (Fig. 5b–f). Proteins were selected as representative of each potentially altered pathway in response to CSIS and/or Tian treatment. Two-way ANOVA revealed significant main effects of combined impact of Tian × CSIS (F_1.19_ = 7.40, p < 0.01) in regard to α-tubulin expression changes with significant increase in CSIS rats (1.60-fold change (fc), ^*^p < 0.05) and Tian-treated Controls (1.73 fc, ^**^p < 0.01). For Cyt c expression, ANOVA revealed significant main effect of combined impact of Tian × CSIS (F_1.20_ = 5.32, p < 0.05), whereby a significant increase in expression was observed in CSIS rats (^*^p ≤ 0.05), which was, opposite to results of proteomic study, reversed by Tian treatment (^^^p < 0.05). For HSP90 expression changes, two-way ANOVA failed to detect any significant main effects or significant difference in expression changes between the examined groups.

ANOVA revealed no significant main effects in regard to Glud1 expression changes. However, the trend of up-regulated protein expression was observed in Tian-treated CSIS rats. For Glul expression changes, CSIS showed a significant main effect as revealed by ANOVA (F_1.20_ = 9.44, p < 0.01). Glul was significantly increased in CSIS rats (1.28 fc, ^**^p < 0.01), as detected by proteomic study. The same trend was observed in Tian-treated CSIS rats (^*^p < 0.05).

## Discussion

Many molecular alterations associated with the pathophysiology and severity of MDD as well as the effects of the fast acting antidepressants reside within the synapse^47,48^. To reveal the molecular basis of MDD and effects of Tian, hippocampal synaptoproteome changes in rats exposed to CSIS with or without antidepressant Tian treatment was done using 1D SDS-PAGE in combination with a nano LC–MS/MS system analysis. The present study was conducted on the animals that underwent behavior assessment and were classified according to behavior parameters resembling depressive- and anxiety-like behaviors and antidepressant efficacy^14^. Briefly, 6 weeks of CSIS induced a significant decrease in SP, an increase in the number of buried marbles and an increase in immobility time in the FST, thus suggesting the CSIS-induced state of anhedonia, anxiety and behavioral despair in rats, which are all features of depressive- and anxiety–like behaviors. Administration of Tian (10 mg/kg/day) to CSIS rats for 3 weeks reversed these effects, thus causing the normalization of SP, number of buried marbles and immobility behavior in rats, thus showing its antidepressant- and anxiolytic-like effect.

We previously demonstrated that Tian triggered the sub-proteome changes in hippocampal cytosol and NSM of both control and CSIS rats, whereby up-regulated energy-related proteins in NSM of controls and CSIS rats probably lead to the nootropic and antidepressant effects, respectively^14^. Nonetheless, Tian’s antidepressant efficacy has been also shown on various animal model systems for depression^38,49–52^. Regarding used behavior tests, SP and MB represent non-invasive mild tests, while FST has been related with some neurochemical changes^53^. Given that our results are a relative representation of subproteome changes, and that all animals were subjected equally to same behavioral testing, we supposed that any possible effect of behavioral testing is relativized. Also, not until at least 24 h after the last test has been performed, the scarification procedure was carried out. Hormone-based implications are well known as the contributing factor for susceptibility to depressive behaviour as well as in response to treatment. Hence, our study was conducted on male Wistar rat only, and the results of this study should be interpreted strictly in regard to male rat population.

The specificity in studying synaptosomes, as a mean for investigating synaptic transmission, relies on the fact that the entire machinery responsible for neurotransmitter trafficking, mediated by synaptic vesicles as well as the storage, remains intact^17^. In the present study, proteome pattern changes were found in the hippocampal synaptosomal fraction of Tian-treated controls (Supplementary Table S1). The synaptic vesicles release their content in the synaptic cleft by Ca^2+^-dependent exocytosis upon stimulation. Tian up-regulated the expression of Syt1, which is a Ca^2+^ sensor protein that triggers vesicle release^54^. Up-regulation of the Syn1 which tether synaptic vesicles to the actin cytoskeleton within the presynaptic termina and α-tubulin, representative of the change of cytoskeleton organization, were confirmed/validated by the immunohistochemical and Western blot analysis (Fig. 5a,b). Syn1 is a protein involved in neuronal plasticity and synaptogenesis, with specific role in the regulation of neurotransmitter release and synaptic vesicle exocytosis^55^. Phosphorylation of Syn1 by CaamkI causes a release of synaptic vesicles from the cytoskeleton and increases the number of vesicles available for fusion with plasma membrane. In line with this, Tian increased the expression of Camk2a, a subunit of Camk2 that also phosphorylates Syn1 and is involved in synaptic plasticity and cognitive functions^56,57^. Literature data have shown that Camk2 could be implicated in the mechanism of antidepressant drug action^58,59^. In addition, Tian-induced antidepressant effects are associated with increased phosphorylation level of AMPA receptor subunit GluA1, on both Camk2 and PKA site^60^. The increased expression of Dpysl2, also known as collapsing response mediator protein 2, CRMP2, as a substrate for Camk2, suggests the involvement of this protein in synaptic plasticity^61^. Therefore, Tian-increased expression of proteins such as Syn1 and Camk2a in control rats may be a possible mechanism for its memory-protective effectiveness and point, at least in part, on its nootropic efficacy. Literature also revealed Tian memory enhancing properties in the absence of stress^32^. In addition, increases in expression of a number of proteins involved in synaptic vesicle transport relate Tian with the enhanced synapse dynamics.

It was also observed the impact of Tian on up-regulation of a number of glycolysis-related proteins. Boosting of the energy-metabolism was further supported with an increased expression of mitochondrial energy metabolism-related enzymes, such as Atp5b, Atp5o and Cyt c involved in oxidative-phosphorylation, suggesting increased production of ATP. The trend of increased Cyt c expression was also observed by the Western blot analysis (Fig. 5c). Moreover, increased expression of proteins related to the V-ATPases, which generate electrochemical gradient of protons for the uptake and accumulation of transmitters from cytosol into synaptic vesicles^62^ indicate on an increased ATP production. ATP generated by synaptic vesicle-associated pyruvate kinase (Pkm) is harnessed to transport glutamate into synaptic vesicles^63^. Hence, by stimulating the process of neural transmission in the hippocampus of rats under normal physiological state, Tian may improve cognitive preferences related to its nootropic efficacy. Tian treatment may also exert its role by controlling oxidative stress, given that increased expressions of antioxidant-related enzyme Prdx6, responsible for peroxide removal was found. In addition, it was observed an increased expression of HSP90, which could mediate its protective role in response to increased energy demands in the cells. However, this expression change couldn’t be confirmed by the Western blot analysis (Fig. 5d). However, all these proteome changes induced by Tian in normal physiological state could also relate to side effects of its prolonged use as a cognitive enhancer, rather than its antidepressant efficacy.

CSIS also showed a significant impact on synaptosomal proteome (Supplementary Table S2), mainly by increasing the expression of proteins involved in metabolism, actin cytoskeleton and signaling transduction, that was also found in stress response and MDD^64,65^. The up-regulation of proteins related to glucose metabolism and TCA cycle is suggesting on an increased synaptic energy demands of the CSIS rats. Accordingly, glycolytic-made NADH produced by up-regulated protein expression of MDH2 and Got1, would have increased entry to synaptic mitochondria for oxidation. Up-regulated MDH2 has been found in post-mortem brain tissue of MDD patients using proteomic approach^66^. We recently reported that CSIS resulted in impaired transporter processes between hippocampal cytosol and NSM and decreased levels of proteins involved in TCA and oxidative phosphorylation^67^. Observed differences may indicate on different biological processes specific for sensitivity to CSIS. The up-regulated expression of proteins involved in actin cytoskeleton (Actb, Arpc4, Cfl1) suggests the involvement of structural proteins in processes underlying the CSIS. The up-regulation of Actb and Cfl1, its major binding protein, could relate with the coping mechanism under CSIS conditions, since one of the Cfl roles is in mediating synaptic plasticity via regulating actin filament dynamics^68^. The up-regulation of proteins related to signal transduction, synaptic transitions and vesicle-mediated transport in synapse may indicate on a coping mechanism potentially related with a decline in neurotransmitter content associated with depressive-like behavior. Post-mortem studies testify about increased expression of synapsin proteins in the brain of patients who suffered from MDD^69^. Accordingly, increased levels of the levels of V-ATPase proteins are suggesting an increased synaptic vesicle proton pump activity. The increased expression of Cyt c, also aids a significance to the activation i.e. enhancement of electron transport chain in the synaptic mitochondria, which was also confirmed by the Western blot analysis (Fig. 5c).

The AP-2 complex plays a significant role in recycling process of synaptic vesicles which is the process necessary for sustaining synaptic transmission^70^. Thus, increasing the expression of the Ap2a2 part of AP-2 complex, CSIS probably supports the interneuron exchange. AP-2 complex is associated with clathrin-coated vesicle endocytosis, whereby CSIS also induced an increase in the expression of Snap91, a protein responsible for clathrin binding with the coated vesicles. Tian reversed the expression of these proteins in the CSIS rats (Table 1), thus probably reducing the process of synaptic vesicle recycling/retrieval from synaptic cleft. Other constituents of exocytotic processes in the cells, such as Sinaptotagmin-1 and 2, Syntaxin-1B and Syntaxin-binding protein 1, were up-regulated. This may represent the way cells maintain the level of neurotransmitters for obtaining a normal neurotransmission, and resembles the effects of common antidepressants. However, these could also contribute to MDD-related alterations caused by increased level of glutamate, thus leading to structural alterations of neural terminals and subsequent functional alterations in MDD. Overall, synapse vesicle- and energy-related protein changes are similar to those seen in chronic Tian treatment of Controls. However, CSIS resulted with an increased expression of both exo- and endocytosis related proteins mediated by AP-2 complex, while Tian effects were mainly focused on exocytotic process in controls.

As one of the most abundant metabolites in the brain which is often altered in some neurological diseases, myo-inositol is in recent years being intensively studied as a potential plasma biomarker for MDD^71,72^. Our study revealed that Tian treatment boosted the already increased expression of Inositol-1-monophosphatase (Impa1) in the hippocampus of CSIS rats, which is the enzyme responsible for conversion of myo-inositol phosphate to myo-inositol. These results may indicate on local specific increase of myo-inositol content.

Chronic Tian treatment of the CSIS rats revealed sub-proteome changes which could be related with its antidepressant activity (Supplementary Table S3). The increased energy demands, observed by up-regulation of TCA cycle and oxidative-phosphorylation proteins, are in compliance with an increased expression of transport-involved protein Vdac1, possibly suggesting the reinforcement of mitochondrial transport. The same Tian effect in the CSIS rats was previously observed in the hippocampal NSM fraction^14^. We previously identified modifications of the NSM transporter machinery by the chronic fluoxetine treatment^67,73^. In support of this, up-regulated chaperone proteins Hspe1 and Hspa9, which participate in the right protein folding, transport and assembly of transporter was revealed, indicating positive influence of Tian on mitochondrial dynamics.

We recently demonstrated that CSIS compromises the glutathione redox balance in the hippocampus of rats, targeting Gsto1 enzyme activity^5^. Tian up-regulated the proteins with known neuroprotective roles, Prdx5, Sod_Cu domain-containing protein and Gsto1. Given that oxidative stress is increased in stress-induced depression, by increasing the expressions of antioxidant enzymes, Tian could provide a protection for the cells against oxidative stress in the CSIS-exposed rats. Moreover, these results corroborate our previous findings where chronic treatment with Tian enhances protein expression of Cu–Zn Superoxide dismutase in cytosol fraction of the hippocampus of the CSIS rats^14^. As noted above, similar effect of Tian was seen in controls where expression of Prdx6 was increased thus suggesting on a possible common protective mechanism regardless of the physiological condition.

Several hypotheses have been postulated as a pathophysiological cause of MD. A dysfunction of glutamatergic system and malfunction of the mechanism regulating glutamate clearance and metabolism is related with pathophysiology of depression^74^. Significance of these pathways represents potential common targets for different types of antidepressant. We noted the up-regulation of protein related to the glutamate pathway, such as Glud1 in Tian-treated CSIS rats. The same trend was confirmed by the Western blot, however lacking the statistical significance (Fig. 5e). This enzyme converts L-glutamate into alpha-ketoglutarate, a TCA cycle intermediate^75^. Moreover, depressive-like behavior was observed in GluD1 KO mice^76^, where loss of GluD1 prevents a normal developmental pruning of dendritic spines and leads to a higher number of excitatory synapses and increases in glutamatergic neurotransmission in adulthood^77^. Hence, Tian-induced up-regulation of Glud1 may target TCA cycle in the hippocampus of CSIS rats, having a beneficial impact on the glutamate metabolism in the brain. Our result corroborates a recent study demonstrating an increased level of Glud1 in the prefrontal cortex of prenatally stressed rats chronically treated with tianeptine^38^. On contrary, our previous study showed that Tian causes a decline in Glud1 expression in the NSM^14^. Overall, CSIS showed no impact in Glud1 expression in the NSM, while in synaptosomes CSIS induced it’s increase. A number of evidence indicate that the expression of proteins as well as their degradation is dependent on localized translational machinery in the dendrites, which constitute the synaptosomes^78^. Hence, this divergence could be attributable to different demands of these two-type of mitochondria. Opposite to Western blot failure in validation of Glud1 expression changes, the obtained Western blot results revealed Glul up-regulation following Tian treatment (Fig. 5f). Since, both Glud1 and Glul neutralize the level of neurotoxic glutamate by converting it to alpha-ketoglutarate and glutamine, these Tian-induced sub-proteome changes probably aid significantly to glutamate clearance.

There is some discrepancy by comparing the Tian-induced sub-proteome changes in the CSIS rats with those observed in Controls (Table 2). Opposite to its effect seen in controls, chronic Tian treatment in the CSIS rats decreased the expression of proteins involved in glycolytic process (Aldoc, Eno3, Gpi, Pkm), thus indicating a decreased glycolytic energy yielding pathway. Also, down-regulated were subunits of V-ATPase protein (Atp6v1e1, Atp6v1a, Nsf) that have roles in synaptic vesicle budding and fusion^79^ as well as the expression of cytoskeletal proteins (Tuba1a, Tuba1c, Tuba4a, Tubb2a, Tubb4b, Tubb5, LOC100909441) following Tian treatment of the CSIS rats. On the other hand, Tian seams to stimulate TCA cycle reactions and oxidative phosphorylation, at least judged by the expression of those components involved in generation of the electrochemical gradient. Up-regulation of chaperone proteins and enzymes of antioxidant defense, favor a possible Tian mechanism in boosting the antioxidative defense under the CSIS conditions. Consequently, we may conclude that altered homeostasis of cells exposed to chronic stress may also lead to different effects of Tian. However, a similar pattern of action could be related with modulations of synaptic vesicle dynamics. While in controls Tian treatment stimulates the process of synaptic vesicle exocytosis, Tian reduces the endocytosis process in the CSIS rats. Thus, by modulating these dynamic processes, Tian possibly acts in both states with direct/indirect stimulation of neural transmission based on the synaptic vesicle content secured in the synaptic cleft.

**Figure 6:**
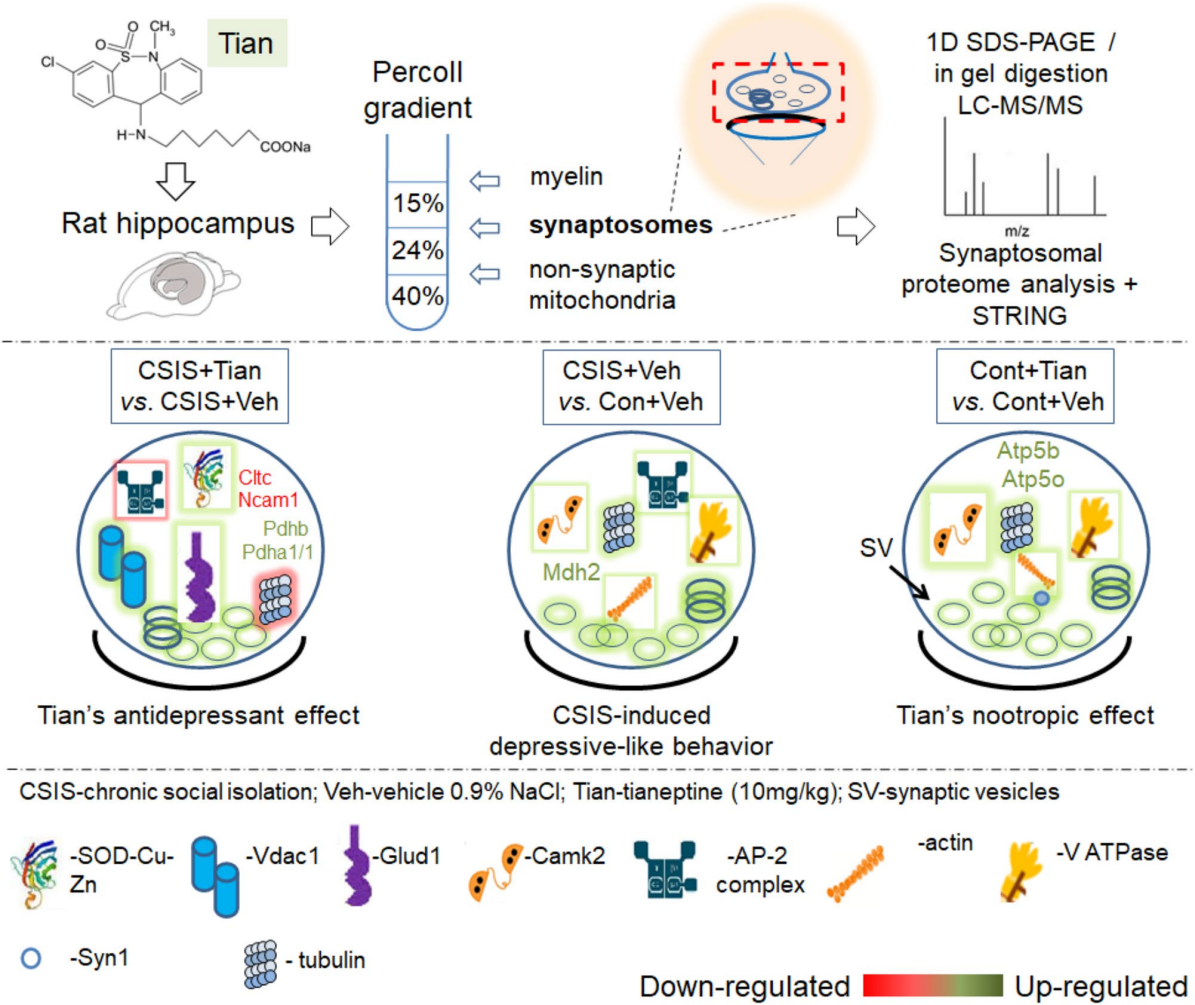
Graphical representation of the obtained proteome changes in rat synaptosomes following CSIS and/or Tian treatment.

In summary, the present study results indicate on a specificity of the synaptoproteome in response to both CSIS and specifically the Tian stimuli (Fig. 6). We suggest potential targets as a useful mechanistic insight into the effects of Tian on synaptic proteins in the CSIS rats, an animal model of depression. Synaptoproteomic analysis of control rats treated with Tian revealed involvement of Syn1 and Camk2-related neurotransmission, vesicle transport and energy metabolism. An increased synaptic energy metabolism of CSIS rats along with up-regulated expression of proteins involved in actin cytoskeleton, signaling transduction and synaptic transmission were revealed. The attenuation of glycolytic pathway accompanied at the same time with the stimulation of the TCA cycle, electron transport chain of the mitochondria, mitochondrial transport and antioxidative defense proteins were recognized as hallmarks of Tian effects in the CSIS rats. The limitation of the present study is the pooling approach applied in the proteomic analysis. Overall, the modulation of synaptic vesicle dynamics and up-regulation of the proteins involved in energy metabolism suggests them as potential targets for Tian treatment and potentially crucial for the effective treatment of stress-related MD.

## Supplementary Information

Supplementary Information 1.

Supplementary Information 2.
