# Supplementary material for: Tianeptine modulates synaptic vesicle dynamics and favors synaptic mitochondria processes in socially isolated rats
Source: Sci Rep. 2021 Sep 7;11:17747. doi: 10.1038/s41598-021-97186-7 (PMC8423821; doi:10.1038/s41598-021-97186-7)

Tianeptine modulates synaptic vesicle dynamics and favors synaptic mitochondria processes in socially isolated rats

Ivana Perić[1], Victor Costina[2], Snežana Djordjević[3], Peter Gass[4], Peter Findeisen[2], Dragoš Inta[5], Stefan Borgwardt[6], Dragana Filipović[1]*

[1]Department of Molecular biology and endocrinology, "VINČA", Institute of Nuclear Sciences - National Institute of the Republic of Serbia, University of Belgrade, Belgrade, Serbia

[2]Institute for Clinical Chemistry, Medical Faculty Mannheim of the University of Heidelberg, University Hospital Mannheim, 68159 Mannheim, Germany

[3]Poisoning Control Centre, Military Medical Academy, Belgrade, Serbia

[4]Department of Psychiatry and Psychotherapy, Central Institute of Mental Health, Medical Faculty Mannheim, Heidelberg University, Mannheim 68159, Germany

[5]Department of Psychiatry (UPK), University of Basel, Basel, Switzerland

[6]Department of Psychiatry and Psychotherapy, University of Lübeck, Lübeck, Germany

* Corresponding author

Supplementary Figure S1. Full-length blots represented in the Figure 5b-f.

WB1

Cont	Cont Tian	CSIS	CSIS Tian	Cont	Cont Tian	CSIS	CSIS Tian

HSP90 – part of the membrane ~ 70-250 kD

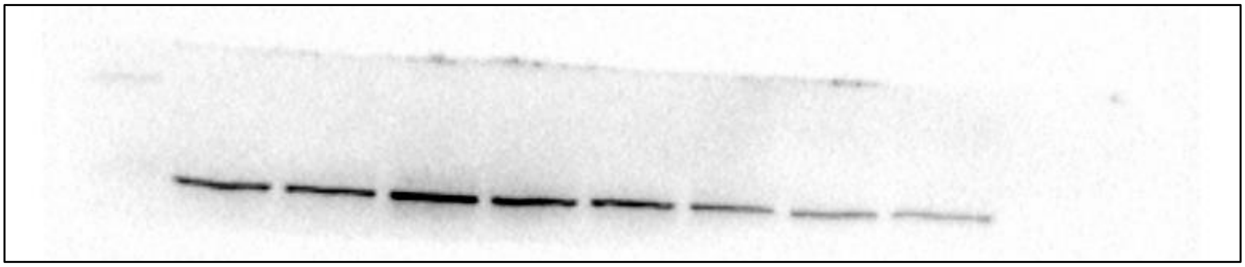

α-tubulin – part of the membrane ~ 50-70 kDa

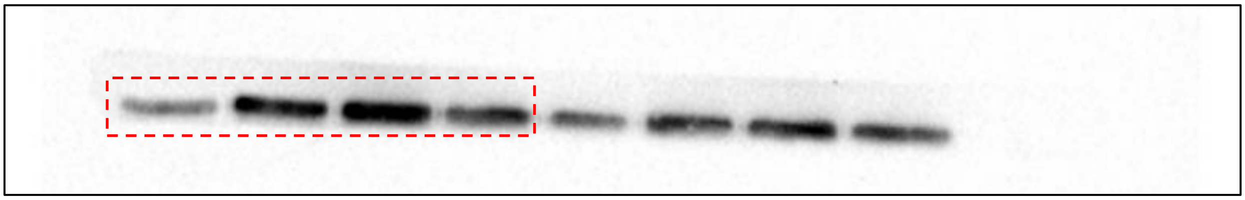

Glul – part of membrane ~ 35-50 kDa

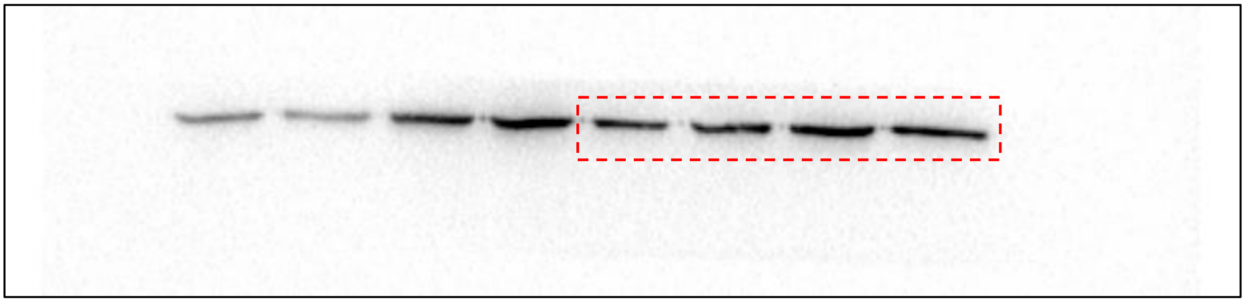

Cyt c – part of the membrane ~ 0-35 kDa

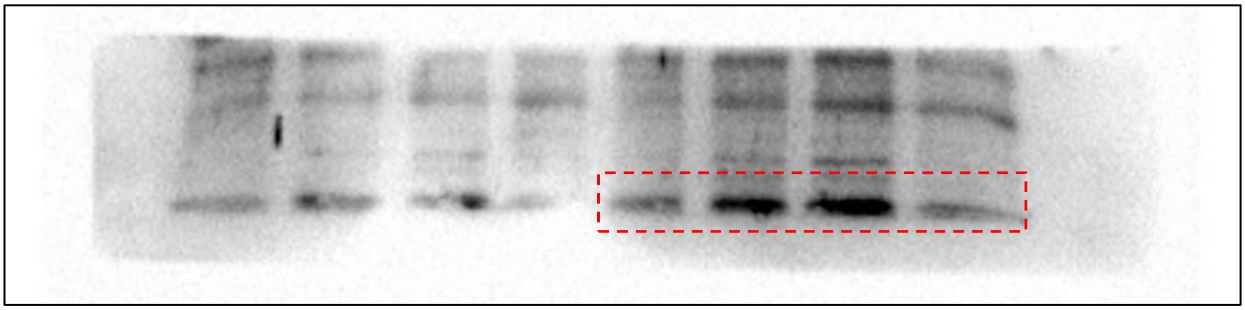

Ponceau S staining

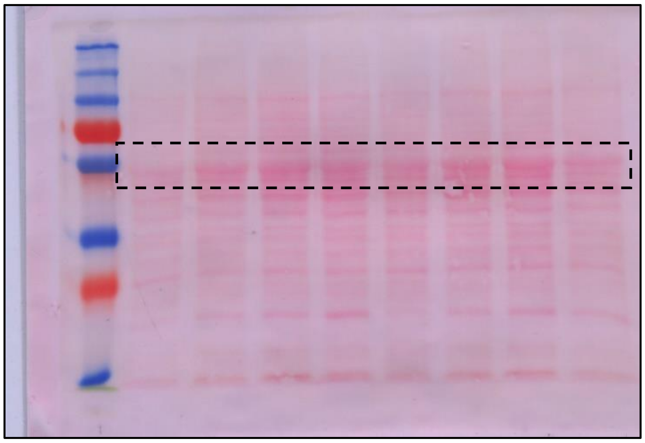

WB 2

Cont	Cont	CSIS	CSIS	Cont	Cont	CSIS	CSIS
	Tian		Tian		Tian		Tian

α-tubulin - part of the membrane ~ 50-70 kDa

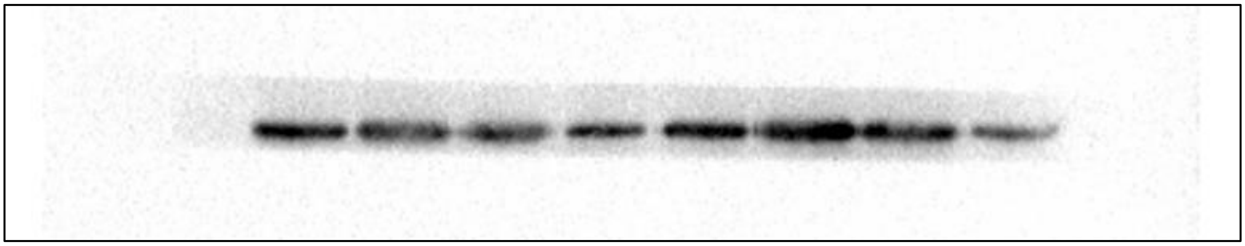

Glud 1 – part of the membrane ~ 50-70 kDa

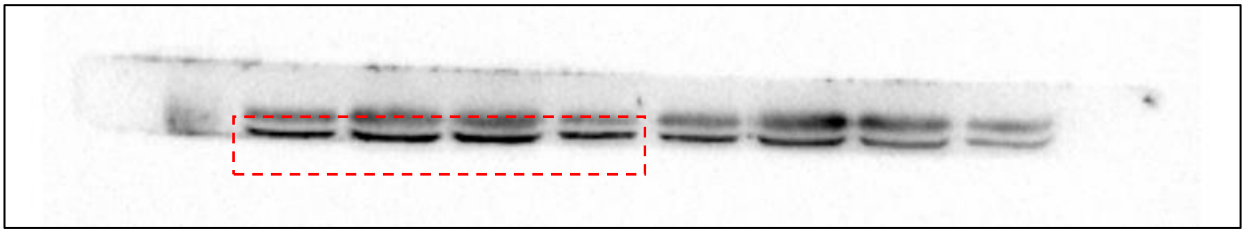

Cyt c - part of the membrane ~ 0-35 kDa

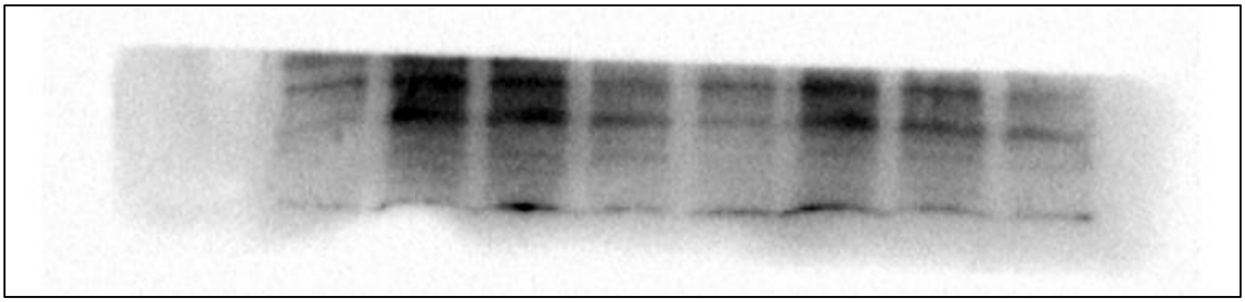

Ponceau S staining

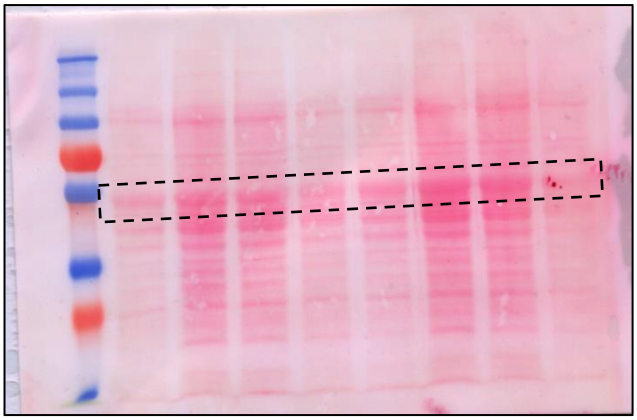

WB 3

Cont	Cont Tian	CSIS	CSIS Tian	Cont	Cont Tian	CSIS	CSIS Tian

α-tubulin - part of the membrane ~ 50-70 kDa

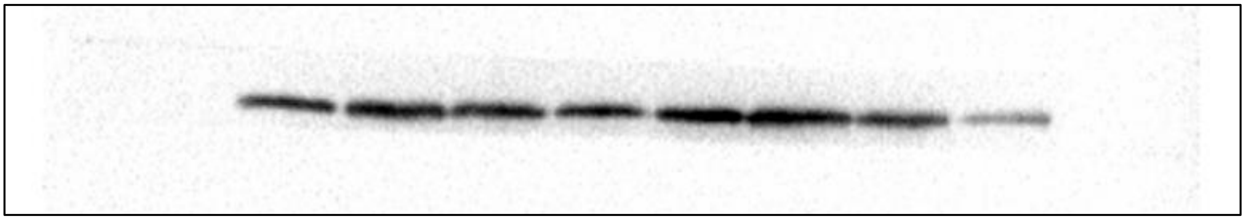

Glud1 - part of the membrane ~ 50-70 kDa

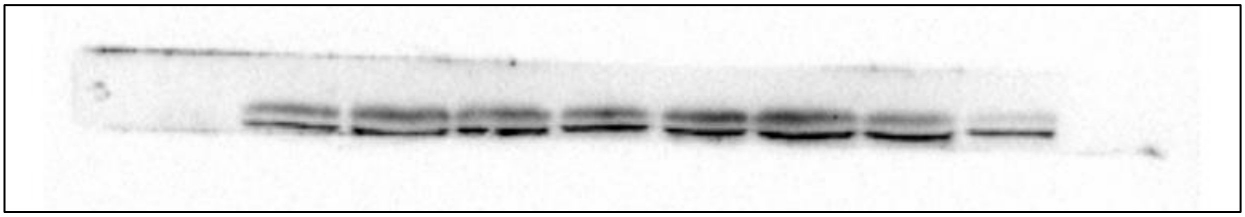

Cyt c - part of the membrane ~ 0-35 kDa

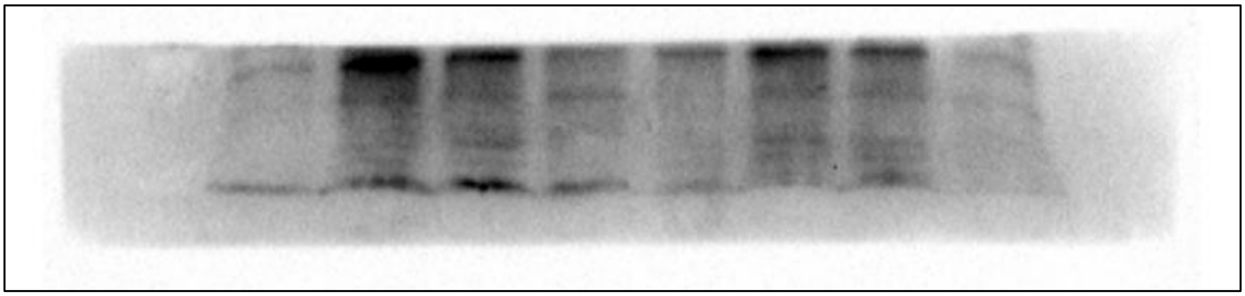

Ponceau S staining

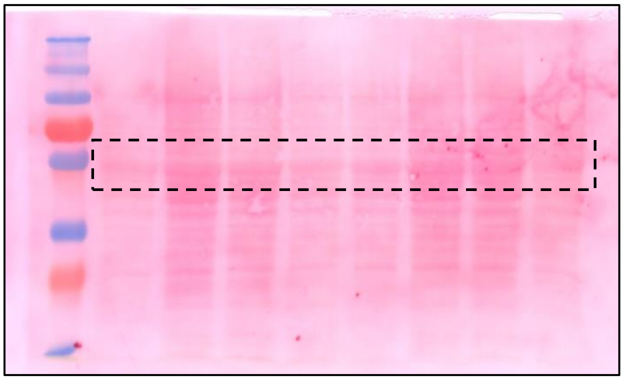

WB4

Cont Cont CSIS CSIS Cont Cont CSIS CSIS

Tian Tian Tian Tian

HSP90 – part of the membrane ~ 70-140 kD

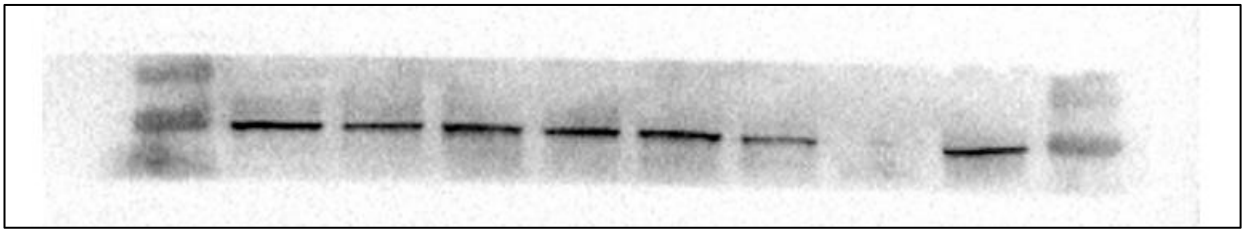

Glud1 - part of the membrane ~ 50-70 kDa

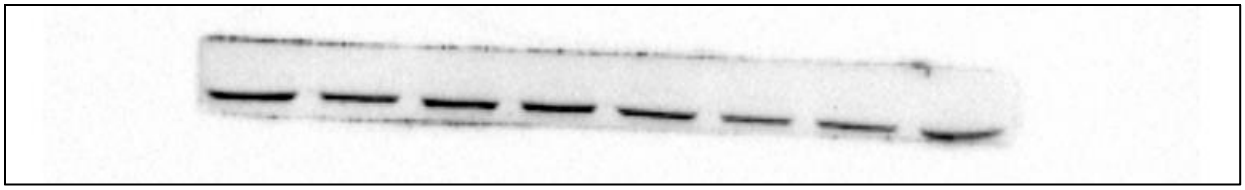

Glul - part of the membrane ~ 35-50 kDa

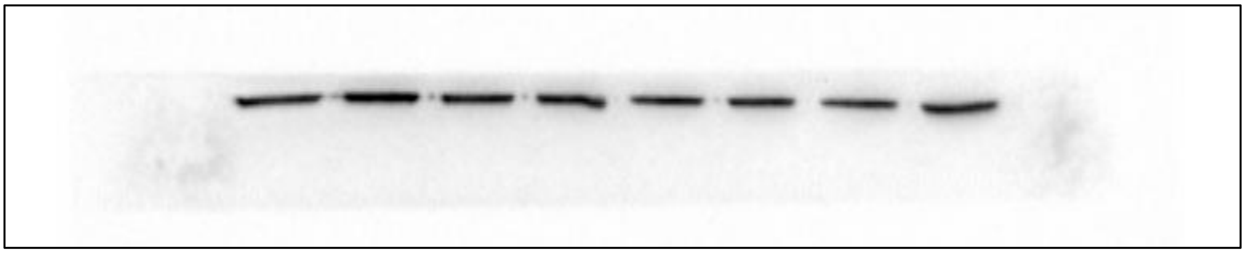

Ponceau S staining

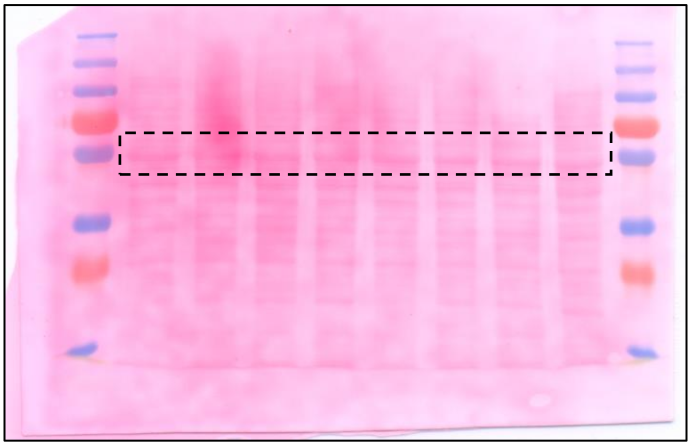

WB5

Cont	Cont Tian	CSIS	CSIS Tian	Cont	Cont Tian	CSIS	CSIS Tian

HSP90 - part of the membrane ~ 70-140 kD

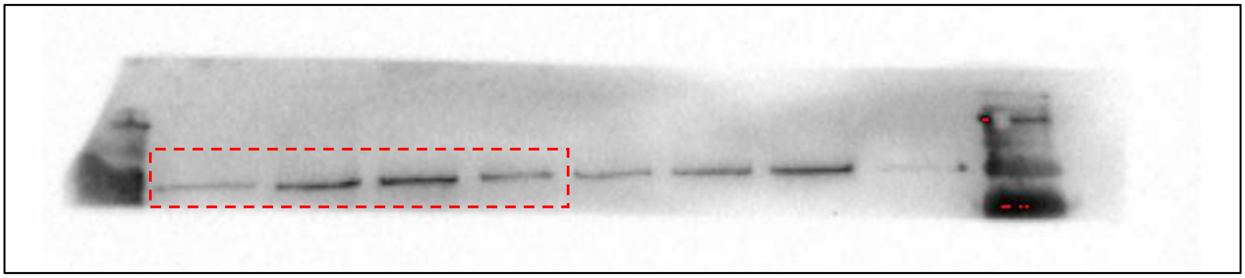

Glul - part of the membrane ~ 35-50 kDa

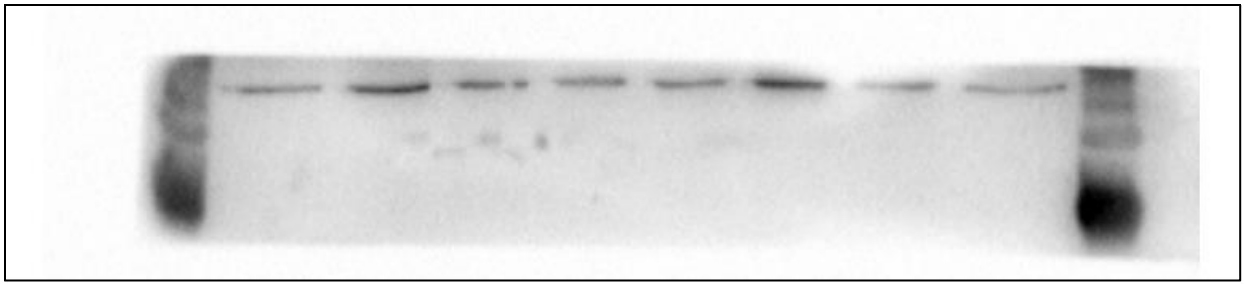

Ponceua S staining

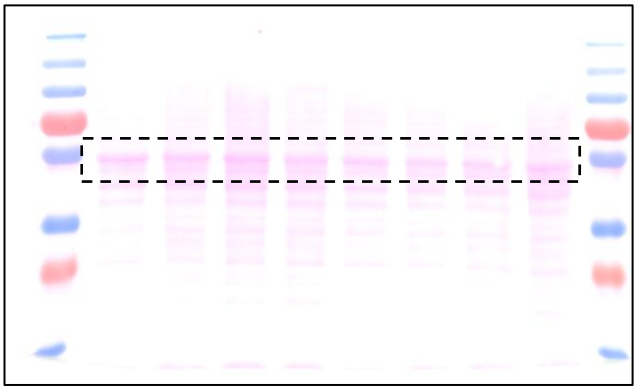

WB6

Cont	Cont Tian	CSIS	CSIS Tian	Cont	Cont Tian	CSIS	CSIS Tian

HSP 90 - part of the membrane ~ 70-140 kD

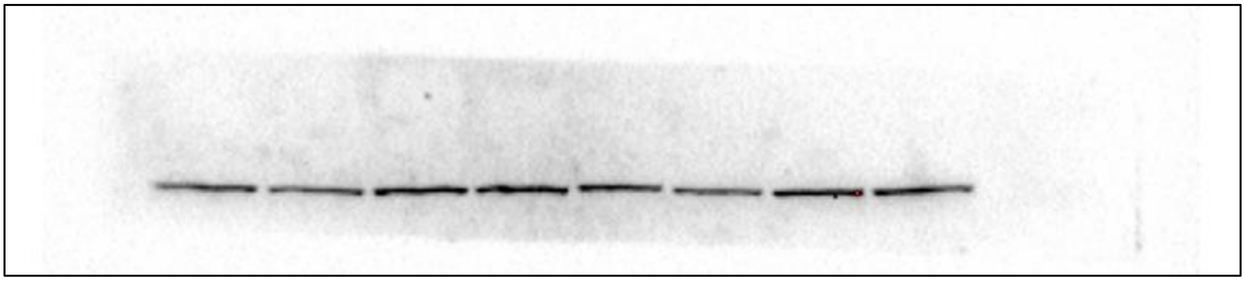

Glul – part of membrane ~ 35-50 kDa

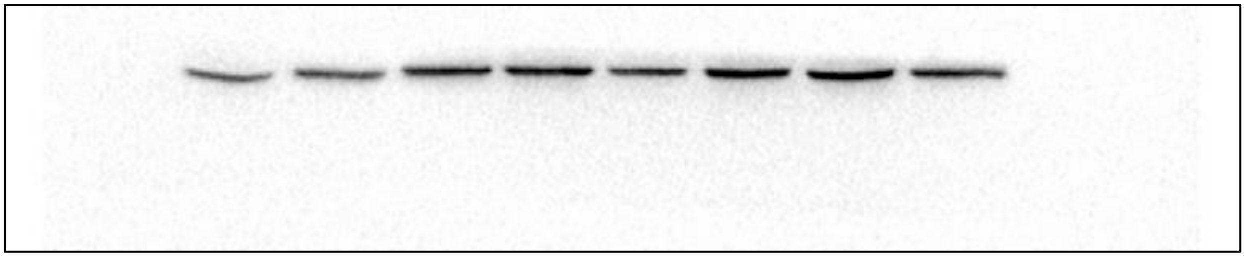

Cyt c - part of the membrane ~ 0-35 kDa

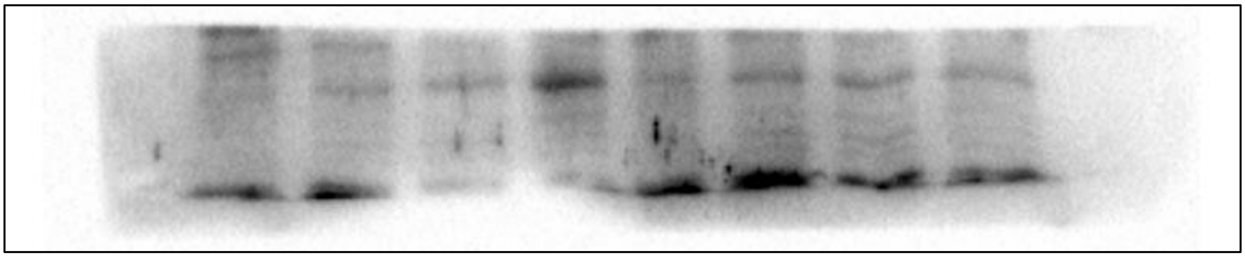

Ponceau S staining

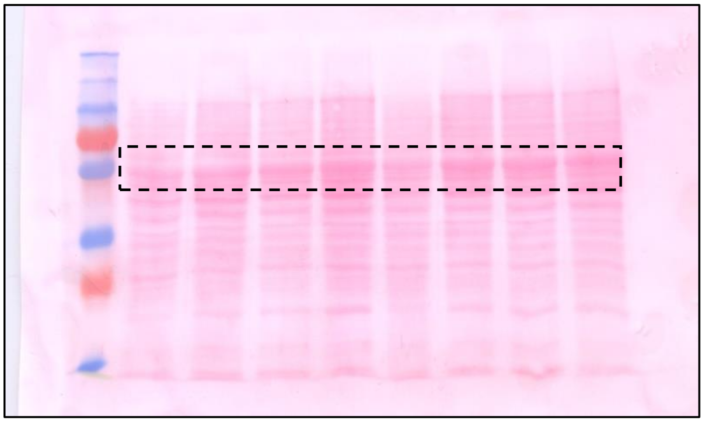

Supplement: Supplementary file 1 — Supplementary Information 1. [file 41598_2021_97186_MOESM1_ESM.pdf]
